# Lysosomal pH dysregulation as a candidate signal-amplifying hub in sepsis-associated lung injury

**DOI:** 10.3389/fcimb.2026.1953667

**Published:** 2026-10-20

**Authors:** Gengjia Tu, Zhijie Deng, Yuling Li, Peigang Tian, Yu Shen, Yanyan Deng, Zhiyuan Zhang, Yi Liu, Shouwei Zhong, Jianjun Wu, Hongquan Zhu, Jiamin Ma, Jianhua Li

**Affiliations:** 1 Department of Critical Care Medicine, The First Affiliated Hospital of Gannan Medical University, Ganzhou, China; 2 The First Clinical Medical College, Gannan Medical University, Ganzhou, China; 3 Department of Emergency Medicine, The First Affiliated Hospital of Dalian Medical University, Dalian, China; 4 Department of Critical Care Medicine, The First Affiliated Hospital of Shihezi University, Shihezi, China; 5 Department of Anesthesiology and Perioperative Medicine, General Hospital of Ningxia Medical University, Yinchuan, China

**Keywords:** acute respiratory distress syndrome, alveolar–capillary barrier, autophagy–lysosome pathway, host–pathogen interaction, inflammasome, lysosomal pH, sepsis, V-ATPase

## Abstract

Sepsis-associated lung injury arises from a dysregulated host response to infection, but the organelle-level mechanisms linking microbial sensing to alveolar–capillary barrier failure remain incompletely resolved. This Review proposes lysosomal pH dysregulation as a candidate, context-dependent signal-amplifying hub rather than a universal initiating event. We synthesize evidence involving V-ATPase-dependent acidification, ClC-7/OSTM1-mediated chloride homeostasis, TMEM175-dependent ion conductance, mTORC1–TFEB signaling, mitochondrial stress, mitochondria–lysosome contact-site proton transfer, and autophagy–lysosome function while distinguishing direct organelle pH measurements from non-equivalent readouts such as membrane permeabilization, cathepsin redistribution, LysoTracker intensity, and autophagic flux. Of 21 formally appraised mechanistic axes, only 2 met Level I criteria; most evidence was Level III or IV and therefore extrapolative or hypothesis-generating. Across macrophages, epithelial cells, pulmonary endothelial cells, and fibroblasts, lysosomal dysfunction may impair pathogen clearance and cellular quality control while amplifying inflammatory signaling and alveolar–capillary barrier injury. We therefore position lysosomal pH homeostasis as a testable mechanistic and stratification axis whose relevance is likely to vary by compartment, cell type, and disease stage.

## 1 Introduction

Sepsis is a heterogeneous clinical syndrome caused by a dysregulated host response to infection, with overlapping hyperinflammatory and immunosuppressive programs (Wang W. and Liu, 2023; Prescott et al., 2026; Singer et al., 2026; Schuurman et al., 2023; Wang et al., 2026). The lung is among the earliest and most frequently injured organs (Park et al., 2019; Qu et al., 2026). A recent meta-analysis estimated that ARDS develops in 34.0% of patients with sepsis (95% confidence interval, 29.0%–39.3%) (Li et al., 2026), and alveolar–capillary barrier failure remains a central pathological feature (Ma et al., 2025; Chan et al., 2025). The cellular processes that convert microbial sensing into persistent pulmonary barrier injury nevertheless remain incompletely resolved.

Endolysosomal organelles occupy a strategic position at the host–pathogen interface. Phagolysosomal acidification supports microbial killing in macrophages; lysosome-related lamellar bodies participate in surfactant biology in type II alveolar epithelial cells; lysosomal degradation and trafficking contribute to endothelial repair; and lysosomal proteolysis supports fibroblast matrix turnover (Di et al., 2006; Pérez-Gil and Frick, 2024; Chintagari et al., 2010). The relevant question is therefore how infection-associated inflammatory and metabolic stress compromises lysosome-dependent functions required for host defense and gas-exchange barrier integrity.

Lysosomal homeostasis depends on V-ATPase-driven proton pumping, counterion transport, membrane integrity, biogenesis, repair, and coordinated autophagic trafficking (Radulovic et al., 2026; Perera and Zoncu, 2016; Mindell, 2012; Wu et al., 2023). We propose that disruption of these layers can create a candidate, context-dependent signal-amplifying hub, not a universal initiating lesion. Throughout the Review, direct measurements of organelle acidity are separated from membrane permeabilization, cathepsin redistribution, hydrolase activity, and autophagic flux, and the evidence is organized within an upstream–midstream–downstream framework (**Figure 1**).

**Figure 1:**
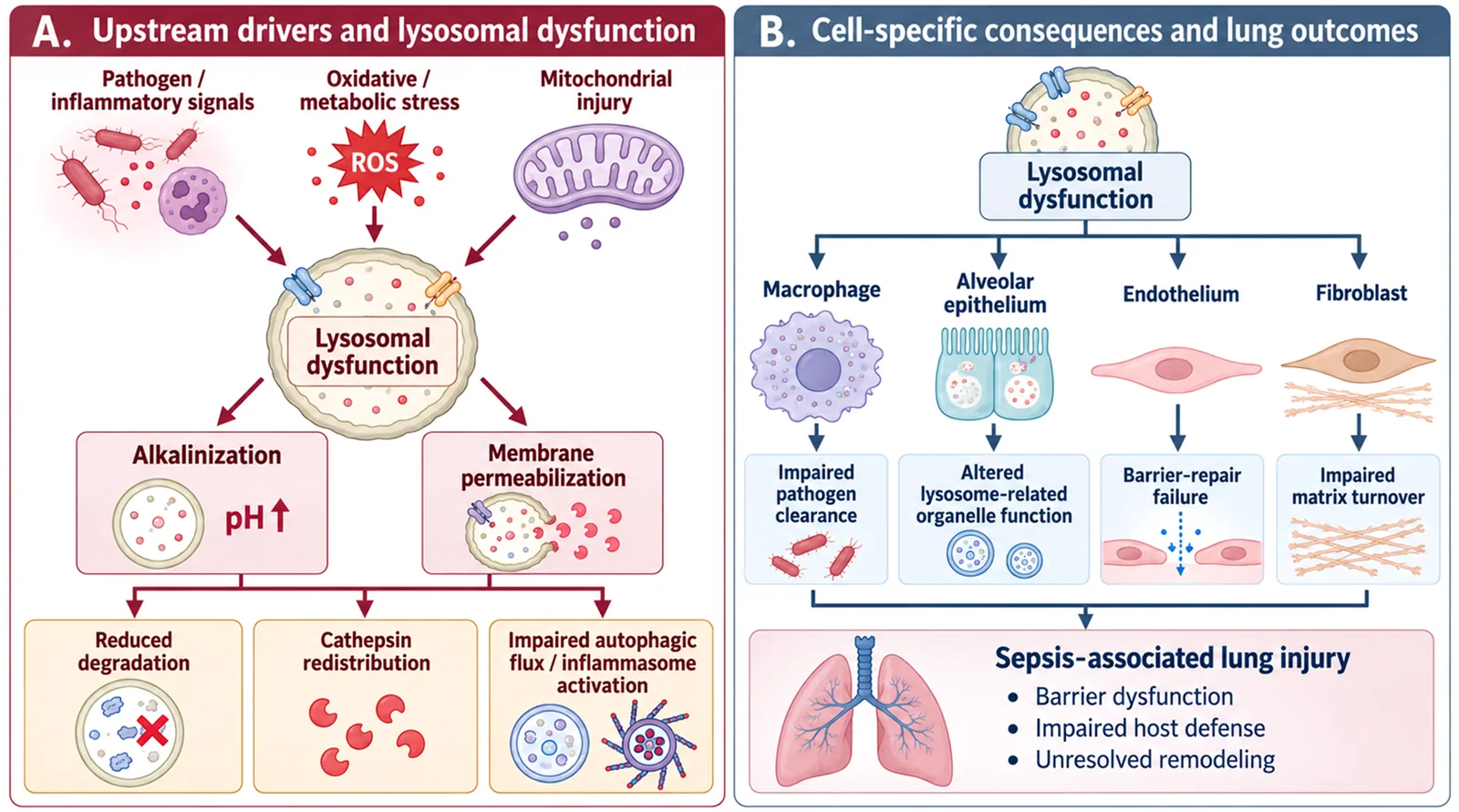
Infection-responsive lysosomal dysfunction as a candidate signal-amplifying hub in sepsis-associated lung injury. **(A)** Upstream drivers, including pathogen-associated and host-derived inflammatory signals, oxidative and metabolic stress, and mitochondrial injury, can disturb lysosomal acidification, ionic homeostasis, membrane integrity, and biogenesis. Alkalinization and membrane permeabilization are distinct midstream defects that can reduce degradation, promote cathepsin redistribution and inflammasome activation, and impair autophagic flux. **(B)** Cell-specific consequences include defective macrophage pathogen clearance, altered epithelial lysosome-related organelle function, endothelial barrier-repair failure, and impaired fibroblast matrix turnover, collectively contributing to sepsis-associated lung injury. Solid arrows indicate directly supported relationships in the cited model; dashed arrows indicate context-dependent extrapolation; dotted arrows indicate untested pH-dependent hypotheses.

## 2 Literature search and evidence-appraisal approach

Relevant studies were identified through searches of PubMed, Web of Science, Scopus, and Google Scholar using combinations of the terms “sepsis,” “infection,” “acute lung injury,” “ARDS,” “lysosomal pH,” “endolysosome,” “phagosome,” “V-ATPase,” “ClC-7,” “TMEM175,” “autophagy,” “inflammasome,” and “mitochondria–lysosome crosstalk.” Primary mechanistic studies were prioritized when they directly measured organelle acidity or causally manipulated lysosomal machinery in sepsis, infection, or lung-relevant models; narrative and systematic reviews were used mainly for background and citation tracing. Studies were excluded from formal evidence-level assignment when they did not inform organelle acidity, ion homeostasis, lysosomal membrane injury, autophagy–lysosome function, or the septic-lung communication axes discussed here. Recent and 2026-dated references were rechecked against PubMed and journal websites on 1 September 2026. Because the original narrative-review search log was not prospectively archived, a formal PRISMA study-flow count cannot be reconstructed reliably; this limitation is stated explicitly in Section 8.5. Evidence was stratified as follows: Level I, direct pH-related measurement with causal intervention in sepsis or sepsis-associated lung injury; Level II, causal lysosomal or autophagy–lysosome evidence in septic lung injury without direct pH measurement; Level III, direct mechanistic evidence from non-septic lung, immune, or other cell models; and Level IV, established intercellular communication in septic lung injury for which pH dependence remains hypothetical. Of 21 formally appraised mechanistic axes, 2 (9.5%) were Level I, 3 (14.3%) Level II, 11 (52.4%) Level III, and 5 (23.8%) Level IV (**Table 1**). Unless otherwise stated, Level III findings are treated as mechanistic extrapolations and Level IV pH dependencies as hypothesis-generating checkpoints. Detailed study-level appraisal is provided in **Supplementary Table S1**, and proposed intercellular checkpoints are summarized in **Supplementary Table S2**.

**Table 1:** Main-text evidence map for lysosomal pH-related mechanisms discussed in this Review.

Evidence level	n/N (%)	Representative mechanistic axes	Interpretation/principal limitation
Level I	2/21 (9.5%)	Circulating mtDNA–STING–TBK1; ZLN005–PGC-1α–TFEB–PI3K/V-ATPase	Direct pH-related measurement with causal intervention in sepsis/sepsis-associated lung injury; evidence remains concentrated in selected phagocyte/systemic models.
Level II	3/21 (14.3%)	PICK1-dependent lysosomal competence; SIRT1–Rab7 late-endosomal mitophagy; cathepsin B–pyroptosis	Causal lysosomal/autophagy–lysosome evidence in septic lung injury without direct quantitative lysosomal pH measurement.
Level III	11/21 (52.4%)	ATP6V0D2; CFTR; lamellar-body V-ATPase; ClC-7/OSTM1; TMEM175; AKT–V-ATPase; TFEB; V-ATPase B2; NHE9 cargo triage; lysosomal impairment–EV release; mitochondria–lysosome contact-mediated proton transfer	Direct mechanistic pH/ion/trafficking evidence, but primarily from non-septic, non-pulmonary, or compartment-distinct models; extrapolation to septic lung requires validation. Contact-site proton-transfer evidence is direct but currently derives from non-septic models.
Level IV	5/21 (23.8%)	Epithelial→macrophage EVs; macrophage→epithelial/endothelial signals; endothelial→myeloid EVs; neutrophil→endothelial signals; fibroblast–macrophage coupling	Communication pathways are established in septic lung injury, but dependence on lysosomal pH remains hypothetical.

Counts refer to the 21 mechanistic axes formally appraised in **Supplementary Table S1**, not to every individual citation or downstream pathway mentioned in the text. Full study-level evidence and limitations are provided in **Supplementary Table S1**.

## 3 Lysosomal pH regulation in infection-relevant lung cells

### 3.1 V-ATPase as an acidification and signaling platform

Lysosomes are the principal acidic organelles for intracellular degradation and recycling, and lysosomal hydrolases generally require V-ATPase-dependent luminal acidification (Mindell, 2012; Suzuki et al., 2023). V-ATPase contains a cytosolic V1 ATP-hydrolyzing domain and a membrane V0 proton-translocating domain. In lung cells it supports both canonical lysosomal degradation and specialized vesicular functions. For example, the C2-a subunit has been localized to lamellar bodies in type II alveolar epithelial cells, supporting surfactant-vesicle biology without establishing a lung-specific requirement for canonical lysosomal acidification (Chintagari et al., 2010; Marshansky and Futai, 2008; Sun-Wada et al., 2003).

V-ATPase function is regulated by nutrient sensing, complex assembly, trafficking, and stress. mTORC1 and TFEB influence lysosomal adaptation, while macrophage ATP6V0D2 facilitates autophagosome–lysosome fusion and restrains inflammasome activation during bacterial challenge rather than simply setting basal lysosomal pH (Settembre et al., 2011; Martina et al., 2012; Xia et al., 2019). V-ATPase is therefore treated as an acidification and signaling platform, not an isolated driver of lung injury.

### 3.2 Counterion pathways and compartment-specific ion homeostasis

The establishment and maintenance of an acidic lysosomal lumen require coordination between electrogenic proton pumping and counterion transport (**Figure 2**). V-ATPase pumps H^+^ into the lumen, generating a positive membrane potential that can limit further proton entry unless charge is dissipated by anion influx, cation efflux, or coupled antiport (Mindell, 2012). ClC-7 is a late-endosomal and lysosomal Cl^-^/H^+^ exchanger that requires OSTM1 for stability and function (Kasper et al., 2005; Kornak et al., 2001; Jentsch and Pusch, 2018; Accardi, 2021).

**Figure 2:**
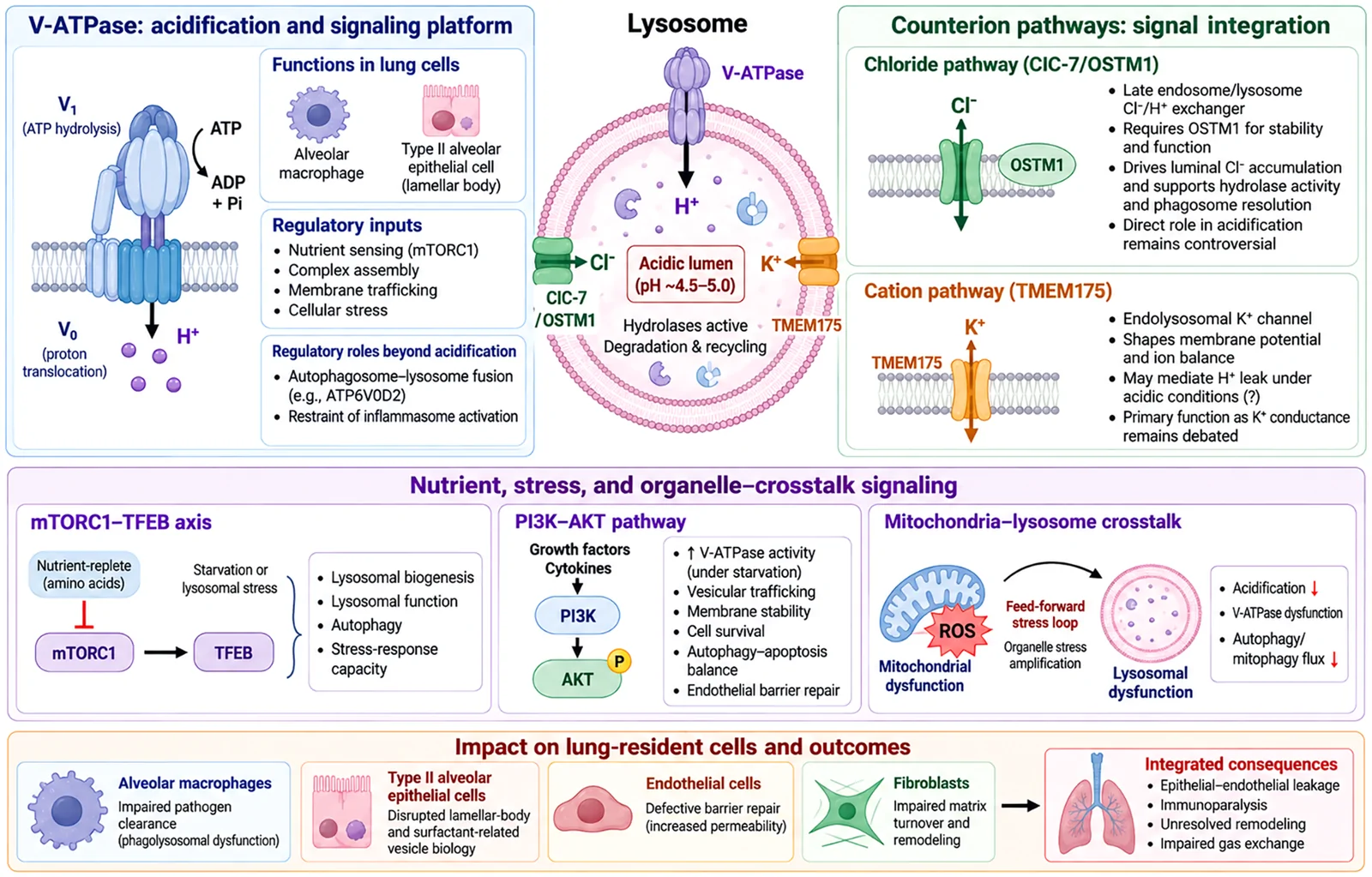
Compartment-specific control of lysosomal pH and function in lung-relevant cells. V-ATPase-dependent proton pumping is coordinated with ClC-7/OSTM1-mediated chloride homeostasis, TMEM175-dependent cation conductance, nutrient and stress signaling, and mitochondria–lysosome crosstalk. The figure distinguishes canonical lysosomes from phagolysosomes and lysosome-related organelles. ClC-7 is presented primarily as a regulator of luminal chloride and hydrolase function, whereas the ion selectivity and pH contribution of TMEM175 remain context-dependent. Effects on macrophages, epithelial cells, endothelial cells, and fibroblasts require cell-specific validation in infection models.

Recent primary studies show that ClC-7 drives luminal chloride accumulation in mature phagosomes and supports hydrolase activity and phagosome resolution (Wu et al., 2023). However, whether ClC-7 directly determines lysosomal acidity remains controversial. Several genetic studies have reported altered chloride homeostasis and lysosomal storage phenotypes without consistent impairment of luminal acidification, suggesting that ClC-7 may primarily use the V-ATPase-generated pH gradient to build luminal chloride rather than simply enabling acidification (Accardi, 2021). We therefore avoid describing ClC-7 as the predominant chloride channel in lung lysosomes unless lung-specific primary evidence is available. Instead, we present it as a key regulator of lysosomal chloride homeostasis with context-dependent effects on pH.

In addition to chloride pathways, lysosomal cation channels can influence pH stability by shaping membrane potential and ion balance. TMEM175 is an endolysosomal potassium channel implicated in lysosomal membrane potential and pH stability (Lee et al., 2017). Its precise ion selectivity remains debated. One model proposes proton conductance under acidic conditions, whereas subsequent work supports predominant K^+^ conductance and argues against a physiologically important proton-selective leak (Hu et al., 2022; Riederer et al., 2026; Zheng et al., 2022). Because these data derive largely from non-septic, neurodegeneration-related or heterologous models, TMEM175 is treated here as Level III evidence and not as an established component of septic pulmonary lysosomal dysfunction. Direct testing in alveolar macrophages, epithelial cells, and pulmonary endothelial cells during sepsis remains necessary.

### 3.3 Nutrient, stress, and organelle-crosstalk signaling

#### 3.3.1 mTORC1–TFEB signaling

The mTORC1–TFEB axis links nutrient sensing to lysosomal biogenesis, autophagy, and stress adaptation. mTORC1-dependent phosphorylation regulates TFEB localization, whereas starvation or lysosomal stress can promote TFEB nuclear translocation and coordinated lysosomal gene expression (Settembre et al., 2011; Martina et al., 2012; Roczniak-Ferguson et al., 2012; Tan et al., 2022; Takla et al., 2023; Cui et al., 2023). TFEB should not, however, be assumed to upregulate every acidification-related transporter across all contexts; its effects on individual V-ATPase subunits, chloride/proton exchangers, and other ion transporters remain cell- and stimulus-dependent (Kim et al., 2024; Wang B. et al., 2024; Akayama et al., 2025).

#### 3.3.2 PI3K–AKT signaling

The PI3K–AKT pathway can modulate lysosomal adaptation through metabolism, V-ATPase activity, trafficking, and membrane stability (Collins et al., 2020; Chen et al., 2025; Song et al., 2020). In non-septic mammalian cells, AKT activation increases V-ATPase-dependent acidification during amino-acid starvation (Ratto et al., 2022). In septic lung injury, PI3K–AKT dysregulation may influence lysosomal resilience indirectly through mitochondrial metabolism, autophagy, cell survival, and endothelial repair, but a direct AKT-driven lysosomal alkalinization mechanism has not been established (Huang et al., 2024; Hu et al., 2024; Zhang et al., 2024).

#### 3.3.3 Mitochondria–lysosome crosstalk

Mitochondria and lysosomes form a bidirectional stress-response network through mitophagy, ROS, metabolic signaling, lipid trafficking, and membrane contact sites (Kiraly et al., 2025). Mitochondrial injury can reduce ATP availability and disturb redox homeostasis, potentially limiting V-ATPase-dependent acidification; conversely, defective lysosomal acidification or trafficking can impair mitophagy and promote accumulation of dysfunctional mitochondria (Liu Q. et al., 2021; Kim et al., 2025; Lo et al., 2024). This feed-forward model is biologically plausible in sepsis-associated lung injury, but direct mapping of lysosomal pH–mitochondrial coupling across lung-resident cells remains incomplete and is therefore treated as a Level III-informed research priority rather than an established septic-lung pathway (Han et al., 2025; Li S. et al., 2025).

Recent studies have identified a more direct contact-site mechanism that may contribute to lysosomal acidification. (Tian et al., 2026) observed proton transfer from mitochondria to lysosomes at mitochondria–lysosome contact sites and showed that experimentally enforced organelle proximity enhanced lysosomal acidification and lysosomal cargo degradation. (Liu Q. et al., 2026) further demonstrated across yeast and human cell systems that protons generated by the mitochondrial electron transport chain can support vacuolar or lysosomal acidification through interorganelle contacts. Disruption of these contacts during aging or cellular senescence was associated with impaired acidification, whereas engineered tethering restored acidification and enhanced autophagic flux. Collectively, these findings suggest that changes in lysosomal pH may reflect not only alterations in V-ATPase activity and counterion conductance but also changes in mitochondria–lysosome contact dynamics and mitochondrial proton availability. In sepsis-associated lung injury, mitochondrial dysfunction could therefore influence lysosomal acidification through at least two non-mutually exclusive routes: ATP- and redox-dependent modulation of V-ATPase activity and contact-dependent proton transfer. However, neither study examined sepsis-associated lung injury or sepsis-relevant lung-resident cell models, and there is currently no direct evidence demonstrating that changes in contact abundance, contact duration, or proton-transfer capacity causally alter lysosomal pH in septic lung cells. Accordingly, we classify mitochondria–lysosome contact-mediated proton transfer as Level III evidence and consider it a plausible complementary mechanism that requires direct validation in sepsis-associated lung injury (Tian et al., 2026; Liu Q. et al., 2026).

### 3.4 Distinguishing organelle acidity from related lysosomal readouts

Throughout this Review, “lysosome” denotes canonical terminal degradative organelles; “endolysosomal” is used when late endosomes and lysosomes are not resolved. Phagosomes/phagolysosomes are infection-specialized compartments, and lamellar bodies are lysosome-related organelles. Findings are not transferred automatically between compartments.

Five readouts are kept distinct: increased luminal pH, lysosomal membrane permeabilization, reduced hydrolase activity, impaired autophagic flux, and altered lysosomal mass/biogenesis. LC3-II, p62/SQSTM1, cathepsin abundance, or LysoTracker intensity alone cannot establish alkalinization because each is affected by organelle number, trafficking, membrane damage, or cargo load.

### 3.5 Measuring lysosomal pH and function

Quantitative organelle pH measurement ideally uses compartment-targeted ratiometric probes calibrated *in situ* with ionophores and defined-pH buffers. Single-wavelength acidotropic dyes are useful for localization but are influenced by probe loading, lysosomal mass, membrane potential, and photobleaching and therefore should not be interpreted as stand-alone pH values.

Accordingly, evidence is classified as direct pH evidence only when organelle acidity was measured or a calibrated pH-dependent function was causally interrogated. Membrane permeabilization, protease redistribution, autophagic blockade, or altered trafficking without pH measurement are described as broader lysosomal dysfunction, a distinction that is particularly important in sepsis because cell death, systemic acid–base changes, and altered organelle abundance can confound acidotropic readouts (Mindell, 2012; Wu et al., 2023; Suzuki et al., 2023).

## 4 Microbial and host signals disrupting lysosomal homeostasis

Sepsis-associated lung injury reflects combined microbial and host danger signals, cytokines, recruited inflammatory cells, and metabolic/mitochondrial stress (Cao et al., 2021; Scott et al., 2025; Shen et al., 2024; Qiao et al., 2024). Their lysosomal effects are heterogeneous across pathogens, cell types, disease stages, and organelle compartments.

When adaptive capacity is exceeded, disturbed acidification, ion homeostasis, membrane integrity, repair, and autophagic degradation may convert transient stress into sustained injury (Cao et al., 2021; Shen et al., 2024). The resulting defects can amplify inflammation, impair pathogen clearance, and destabilize epithelial–endothelial repair. Lysosomal alkalinization is therefore framed as a possible amplifier rather than the earliest event in every model (**Figure 3**).

**Figure 3:**
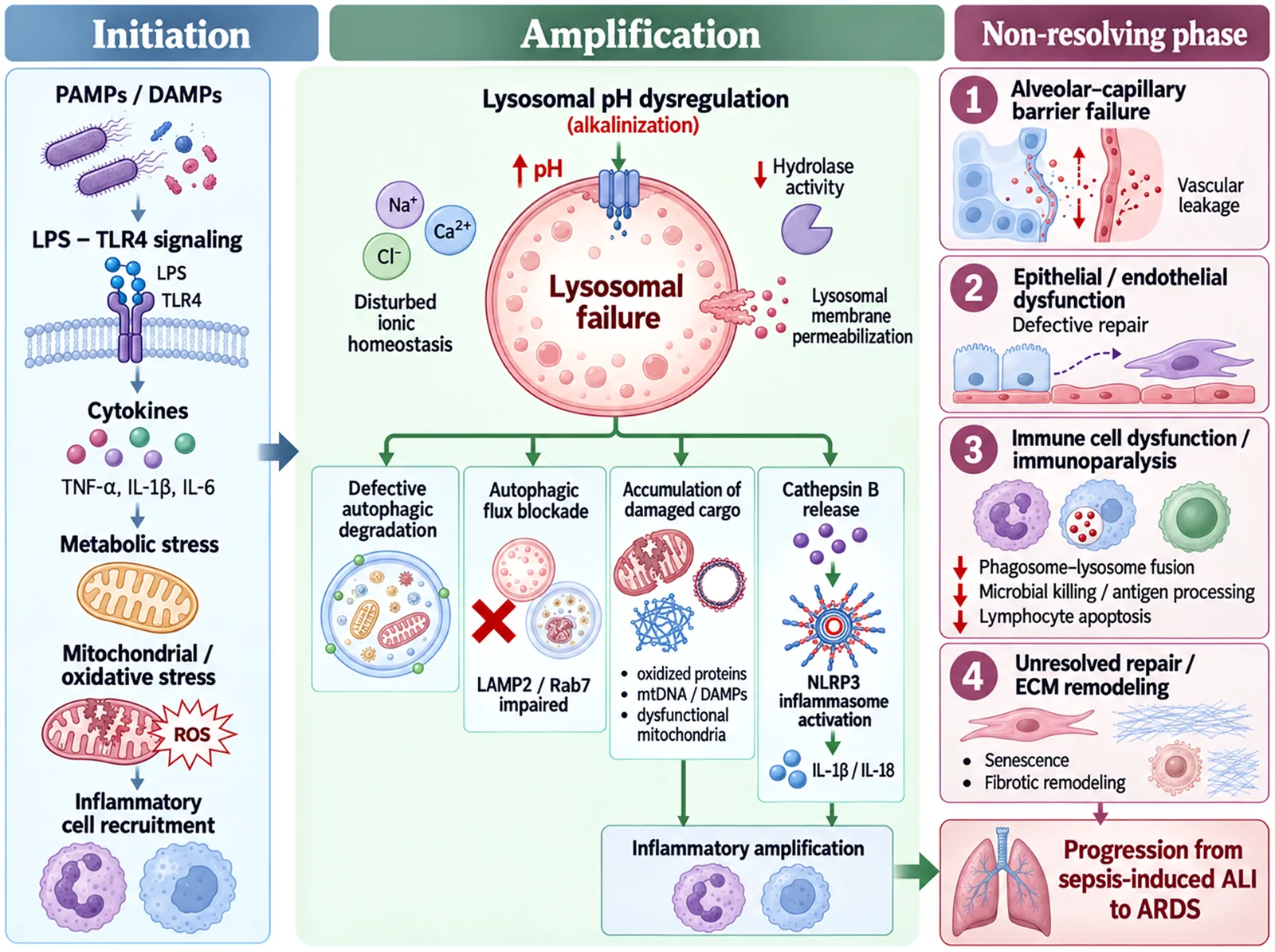
Stage-specific lysosomal dysfunction in sepsis-associated lung injury. During initiation, microbial products, cytokines, ROS, and metabolic stress sensitize lysosomal and autophagic pathways. During amplification, reduced acidity, membrane permeabilization, defective degradation, cathepsin redistribution, inflammasome activation, and mitochondrial injury can form a feed-forward loop. During non-resolution, persistent organelle dysfunction may contribute to impaired pathogen clearance, immunoparalysis, epithelial–endothelial repair failure, senescence, and matrix remodeling. The temporal sequence and dominant cell types remain incompletely defined.

### 4.1 Pathogen-associated and host-derived initiating signals

#### 4.1.1 LPS–TLR4–NF-κB signaling

LPS activates TLR4–MyD88/TRIF signaling and NF-κB-dependent inflammatory transcription in pulmonary immune and structural cells (Kawai and Akira, 2010). Available evidence does not show universal transcriptional repression of lysosomal acidification machinery by LPS. Instead, TLR4/CD14 endolysosomal trafficking and downstream NF-κB/MAPK signaling can indirectly alter cytokine production, nitroxidative stress, metabolism, and autophagy–lysosome flux (Ciesielska et al., 2021; Wang S. et al., 2023).

Persistent LPS stimulation may reduce degradative capacity, impair autophagic clearance, and destabilize lysosomal membranes, thereby facilitating NLRP3 activation through damaged mitochondria, ROS, defective clearance, and lysosome-derived danger signals (Bonacina et al., 2025; Biasizzo and Kopitar-Jerala, 2020). Lysosome-associated scaffolding, including Ragulator/LAMTOR1–HDAC6, further indicates that lysosomes can actively shape inflammasome assembly (Huang et al., 2021; Tsujimoto et al., 2023).

Inflammatory microRNAs may provide an additional regulatory layer. miR-155 is induced by LPS and TNF-α and participates in endotoxin responses (Tili et al., 2007), but no direct evidence currently links miR-155 to lysosomal ion-transporter regulation in septic pulmonary cells. We therefore treat this relationship as an open question rather than part of the core causal model.

#### 4.1.2 Inflammatory cytokines and oxidative-metabolic stress

TNF-α, IL-1β, and IL-6 can promote ROS, mitochondrial injury, metabolic reprogramming, and dysregulated autophagy–lysosome flux during sepsis (Doganyigit et al., 2022; Luo et al., 2022). These convergent stresses may reduce ATP-dependent proton pumping, degradative capacity, ion homeostasis, or membrane stability, but no single cytokine-to-lysosomal-pH pathway is established across septic lung cells (Nagakannan et al., 2020; He et al., 2024).

Subsequent lysosomal stress or membrane permeabilization may facilitate downstream inflammasome activation through cathepsin leakage, damaged-organelle accumulation, mitochondrial ROS, and defective autophagic clearance (Luo et al., 2022; He et al., 2024). NHE6/SLC9A6 regulates endolysosomal maturation and protease activity in non-septic neuronal models (Pescosolido et al., 2021); a proposed IL-6–STAT3–NHE6 connection in septic lung cells has not been directly tested and is considered an open question.

## 5 Cell-specific and stage-specific consequences in the septic lung

### 5.1 Inflammatory amplification and regulated cell-death signaling

Lysosomal alkalinization and lysosomal membrane permeabilization are mechanistically distinct but can cooperate to amplify pulmonary inflammation. Reduced acidity suppresses hydrolase activity and cargo degradation, whereas membrane permeabilization releases cathepsins and other lysosomal contents into the cytosol. Cathepsin B has been implicated in NLRP3 inflammasome activation and pyroptosis-related signaling in experimental sepsis-associated lung injury (Biasizzo and Kopitar-Jerala, 2020; Branco et al., 2022; Xie et al., 2023; Zhang X. et al., 2025). Its contribution varies by cell type and stimulus. Lysosomal dysfunction should therefore be viewed as a permissive amplifier that cooperates with mitochondrial ROS, potassium efflux, DAMPs, and defective autophagic clearance rather than as the sole trigger of inflammasome activation.

Defective lysosome-dependent clearance can reinforce inflammation by allowing damaged proteins, oxidized lipids, mitochondrial fragments, mtDNA, and dysfunctional mitochondria to accumulate as danger signals (Liu Q. et al., 2021; Seoane et al., 2020). These signals can sustain TLR9/cGAS–STING and NLRP3 pathways while dysfunctional mitochondria increase mtROS (Jiang et al., 2024). Thus, reduced lysosomal degradation may couple inflammatory escalation to defective cellular quality control, although the timing and cell-specific contribution of pH changes remain incompletely mapped.

### 5.2 Alveolar epithelial and endothelial barrier failure

Lysosomal dysfunction may contribute to alveolar–capillary barrier failure through protease release, inflammatory signaling, altered trafficking, and defective repair. Cathepsin release and endothelial protease imbalance can promote junctional injury and vascular leakage (Jayawardena et al., 2023; Flemming et al., 2015), but supporting studies do not consistently demonstrate a direct pH-to-junction mechanism. The defensible conclusion is therefore that lysosomal injury can destabilize barrier integrity through convergent proteolytic, inflammatory, and repair defects.

In type II alveolar epithelial cells, acidic lamellar bodies support calcium handling and surfactant secretion, while PICK1 deficiency impairs lysosomal function and aggravates experimental sepsis-associated lung injury (Pérez-Gil and Frick, 2024; Chintagari et al., 2010; Mo et al., 2018). These findings do not establish that canonical lysosomal acidification directly controls surfactant degradation or recycling during sepsis.

### 5.3 Immune-cell dysfunction and immunoparalysis

Lysosomal and phagolysosomal dysfunction can impair macrophage and dendritic-cell antimicrobial activity by reducing microbial killing, cargo degradation, and antigen processing (Weiss and Schaible, 2015; Watts, 2022; Nguyen and Yates, 2021). This is a strong reason to avoid indiscriminate lysosomal inhibition. Quantitative relationships between lysosomal pH, MHC-II expression, and human sepsis-associated immunoparalysis remain insufficiently defined.

Cathepsin leakage can intersect with mitochondrial apoptosis, and lymphocyte depletion contributes to sepsis-associated immunoparalysis (Xie et al., 2023; Wang et al., 2023; Martin et al., 2020; Kox et al., 2026).Direct evidence that restoring lysosomal acidity prevents T-cell apoptosis in human sepsis is lacking; any lysosome-directed immune-recovery strategy must therefore be evaluated alongside established immune checkpoints and metabolic exhaustion.

### 5.4 Autophagic flux blockade and mitochondrial–lysosomal feed-forward injury

Lysosomal pH dysregulation can impair autophagic degradation through reduced hydrolase activity, fusion defects, and Rab7/LAMP2-associated trafficking abnormalities (Li et al., 2013; Zhang et al., 2020; Wright et al., 2016). Because LC3-II accumulation can reflect either induction or degradation failure, it should be interpreted together with p62, lysosomal acidity, and flux assays. Mitochondrial damage and mtDNA–STING signaling provide an additional link between lysosomal dysfunction and inflammatory persistence in sepsis (Liu Q. et al., 2021).

Impaired lysosomal clearance can reduce mitophagy, increase ROS, and further damage lysosomal membranes or ion-transport machinery. This feed-forward mitochondria–lysosome circuit may bridge early inflammatory stress with persistent epithelial–endothelial injury. During non-resolution, defective organelle clearance may also favor senescence and fibrotic remodeling (Qi et al., 2024), but these outcomes should be interpreted as stage-dependent rather than universal consequences of alkalinization.

Recent contact-site studies raise the additional possibility that mitochondrial dysfunction may exacerbate impaired lysosomal acidification by reducing contact-dependent proton transfer, thereby further compromising autophagic degradation and organelle quality control. However, this complementary mechanism has not yet been directly tested in sepsis-associated lung injury (Tian et al., 2026; Liu Q. et al., 2026).

### 5.5 Non-resolving repair and fibrotic remodeling

During late or non-resolving sepsis-associated lung injury, lysosomal dysfunction may contribute to extracellular-matrix accumulation by reducing collagen and elastin turnover, increasing profibrotic inflammatory signaling, and promoting myofibroblast activation. Evidence from pulmonary fibrosis research supports a role for lysosomal acidification in matrix degradation and fibroblast phenotypic regulation (Tian et al., 2025). However, direct human evidence linking lysosomal pH values to post-sepsis fibrotic remodeling remains limited.

### 5.6 Stage-specific synthesis

The proposed network has stage-specific features (**Figure 3**). During initiation, endotoxin, inflammatory cytokines, ROS, and metabolic stress may perturb lysosomal ion balance, membrane integrity, and acidification capacity. During amplification, reduced acidity, hydrolase dysfunction, cathepsin leakage, inflammasome activation, autophagic blockade, mitochondrial injury, and impaired pathogen clearance can reinforce one another (Ratto et al., 2022; Reinheckel and Tholen, 2022; Chevriaux et al., 2020).

During non-resolution, persistent mitochondrial–lysosomal dysfunction may contribute to immunoparalysis, impaired macrophage reprogramming, senescence, defective tissue repair, and matrix remodeling (Tang et al., 2025; Xie et al., 2026).**Figure 3** should therefore be interpreted as a temporal scaffold rather than a fixed linear sequence: the dominant lysosomal phenotype may overlap across phases and vary by cell type, pathogen, treatment, and host response. This stage-based view separates stronger core pathways from emerging mechanisms such as TMEM175-dependent ion conductance and cell-specific mitochondria–lysosome contact-site regulation, including contact-mediated proton transfer.

## 6 Intercellular amplification and signal clearance

Sepsis-associated lung injury is a multicellular process involving alveolar macrophages, epithelial cells, pulmonary endothelial cells, neutrophils, recruited monocytes, lymphocytes, and fibroblasts. Lysosomal dysfunction could influence this network in sender cells by altering cargo degradation and extracellular release and in recipient cells by modifying endosomal processing, mitochondrial quality control, or signal termination. The evidence does not establish lysosomal alkalinization as a universal initiator of these communication pathways; proposed pH dependencies are Level IV unless direct evidence is stated otherwise.

Four processes provide a concise framework (**Figure 4**): cargo triage between intracellular degradation and export; generation or release of extracellular vesicles (EVs), cytokines, proteases, and DAMPs; recipient-cell processing of transferred cargo; and lysosome-dependent termination of extracellular signals through bacterial, debris, apoptotic-cell, or matrix-fragment clearance. Study-level details are retained in **Supplementary Table S2**.

**Figure 4:**
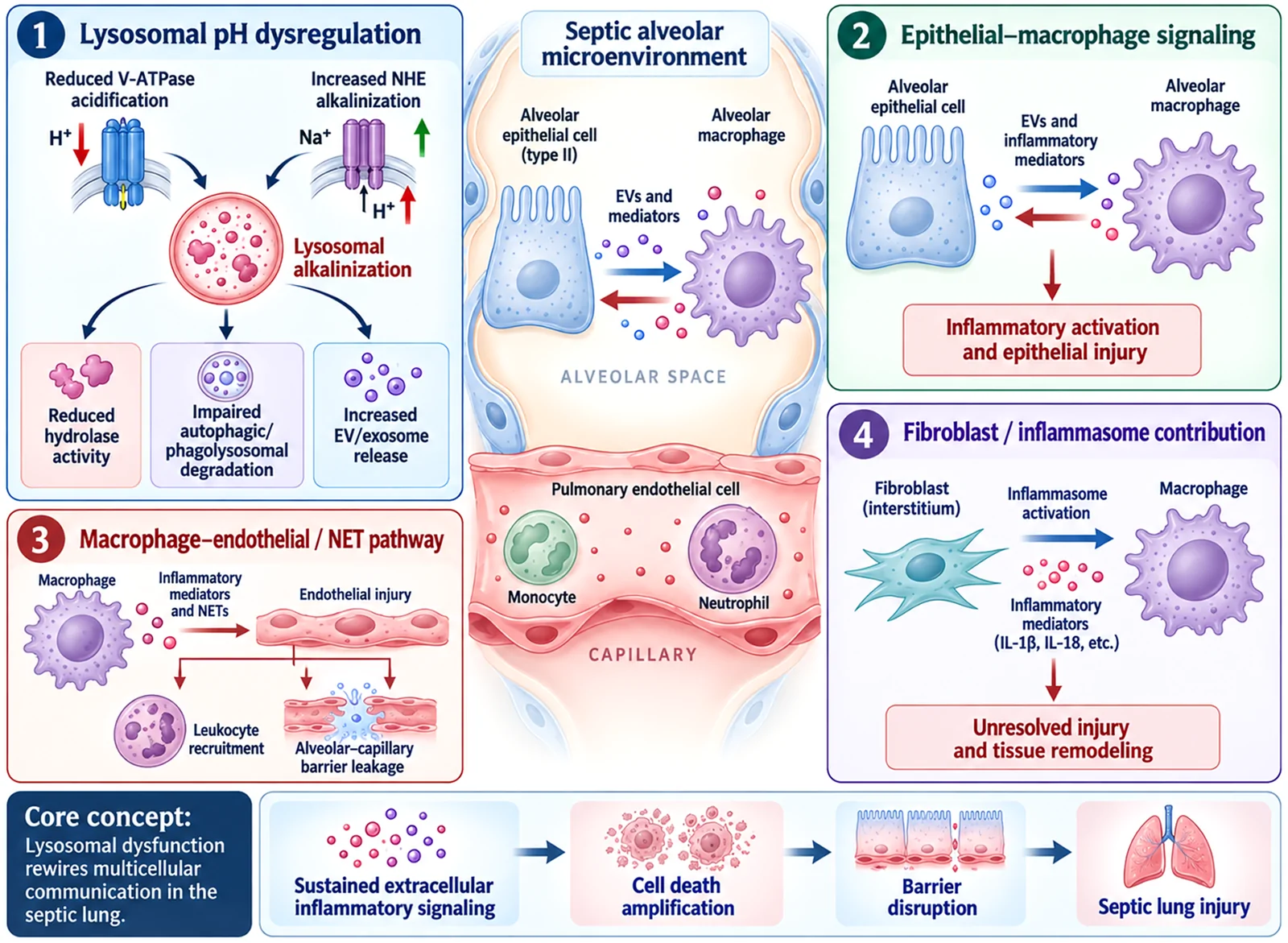
Potential lysosomal checkpoints in multicellular communication in the septic lung. Endolysosomal dysfunction may influence intracellular cargo triage, extracellular vesicle release, recipient-cell cargo processing, mitochondrial quality control, and clearance of extracellular damage signals. Established septic-lung communication pathways include epithelial–macrophage, macrophage–endothelial, endothelial–myeloid, neutrophil–endothelial, and fibroblast–macrophage signaling. Gray dashed connections identify proposed pH-dependent checkpoints that have not been directly tested. Active vesicle secretion should be distinguished from passive cargo release during cell lysis.

### 6.1 Endolysosomal pH may influence the degradative–secretory fate of inflammatory cargo

V-ATPase-dependent acidification supports degradation of endocytic, phagocytic, and autophagic cargo. Late endosomes and multivesicular bodies can instead fuse with the plasma membrane for EV release. In non-septic models, NHE9-mediated endosomal alkalinization impairs Rab7-dependent lysosomal delivery and promotes Rab27-dependent exosome release, whereas pharmacological or trafficking-associated lysosomal impairment increases extracellular release of mitochondrial and ubiquitinated cargo (Duhaini et al., 2025; Liang et al., 2023). These studies support a pH-sensitive degradative–secretory switch but do not establish it in septic lung cells.

This distinction is relevant because active EV secretion, passive cargo release during membrane-disruptive cell death, lysosomal alkalinization, and lysosomal membrane permeabilization are not equivalent processes. In lung epithelial cells, V-ATPase B2 preserves lysosomal acidity and proteolysis and reduces oxidative-stress-associated membrane permeabilization and cathepsin D redistribution (Lee et al., 2022), supporting pH–membrane-stability coupling in a non-septic lung model.

### 6.2 Representative multicellular communication axes

Experimental septic-lung studies establish bidirectional epithelial–macrophage and macrophage–endothelial signaling. Epithelial EV cargo such as miR-92a-3p and tenascin-C can activate macrophage inflammatory programs, whereas epithelial exosomal lncRNA Rmrp promotes macrophage metabolic suppression during later immunosuppression (Liu F. et al., 2021; Gong et al., 2024; Liu et al., 2026). Macrophage-derived CD13 and GBP2-containing EVs can promote epithelial necroptosis or endothelial ferroptosis, respectively (Gong et al., 2022; Li Z. et al., 2025). Endothelial EVs can also reprogram monocytes or neutrophil trafficking through VCAM1- or KPNB1-associated mechanisms (Wang L. et al., 2024; Zi et al., 2024).

Neutrophil-derived NETs and DLL4-positive neutrophils provide additional endothelial injury signals through STING, junctional pathways, and Notch1/ZBP1-associated PANoptosis, while macrophage–fibroblast inflammatory coupling can involve NLRC4-associated pyroptosis (Zhu et al., 2023; Hong et al., 2026; Jin et al., 2025; Zhang Y. et al., 2025). None of these studies directly showed that altering lysosomal pH changes EV abundance, cargo selection, NET persistence, or recipient-cell responses. A more defensible pH-dependent role is therefore modulation of cargo degradation, recipient-cell stress tolerance, or signal clearance. These proposed checkpoints remain hypothesis-generating and are detailed in **Supplementary Table S2**.

## 7 Biomarkers and therapeutic opportunities

Lysosomal biology offers candidate biomarker and host-directed therapeutic opportunities, but translation requires phenotype resolution rather than reliance on a single lysosomal marker. A clinically useful framework must distinguish impaired acidification from membrane injury, capture temporal and cell-type heterogeneity, and demonstrate that lysosomal modulation does not compromise antimicrobial defense.

### 7.1 Biomarkers: from single markers to dynamic panels

We propose two complementary, hypothesis-generating biomarker panels rather than a single-marker strategy. A BALF-focused panel would combine calibrated ratiometric lysosomal pH in recovered alveolar macrophages, intralysosomal cathepsin B/D activity, cytosolic cathepsin redistribution, and extracellular cathepsin or LAMP1/2 in BALF supernatant. A high-pH/low-intralysosomal-hydrolase phenotype with limited extracellular release would favor impaired acidification, whereas cytosolic cathepsin redistribution with increased extracellular cathepsin/LAMP1/2 would favor membrane injury; mixed phenotypes are possible.

A less invasive peripheral-blood panel could combine calibrated lysosomal pH in circulating monocytes, cathepsin activity/localization, and LC3-II/p62-based flux assessment. LC3-II or p62 alone would not be interpreted as evidence of alkalinization because both are influenced by autophagy induction and degradation failure. These panels are proposed as translational test strategies, not validated diagnostic signatures.

Validation should proceed in longitudinal cohorts with sampling anchored to sepsis onset and ARDS evolution. Analytical reproducibility, compartment specificity, association with clinical phenotypes, and incremental value beyond oxygenation indices, lactate, procalcitonin, and inflammatory markers should be tested before any biomarker-guided intervention is considered. Prospective studies should also determine whether BALF and blood measurements identify the same or distinct lysosomal phenotypes. The corresponding BALF/blood sampling and longitudinal validation framework is summarized in **Figure 5**.

**Figure 5:**
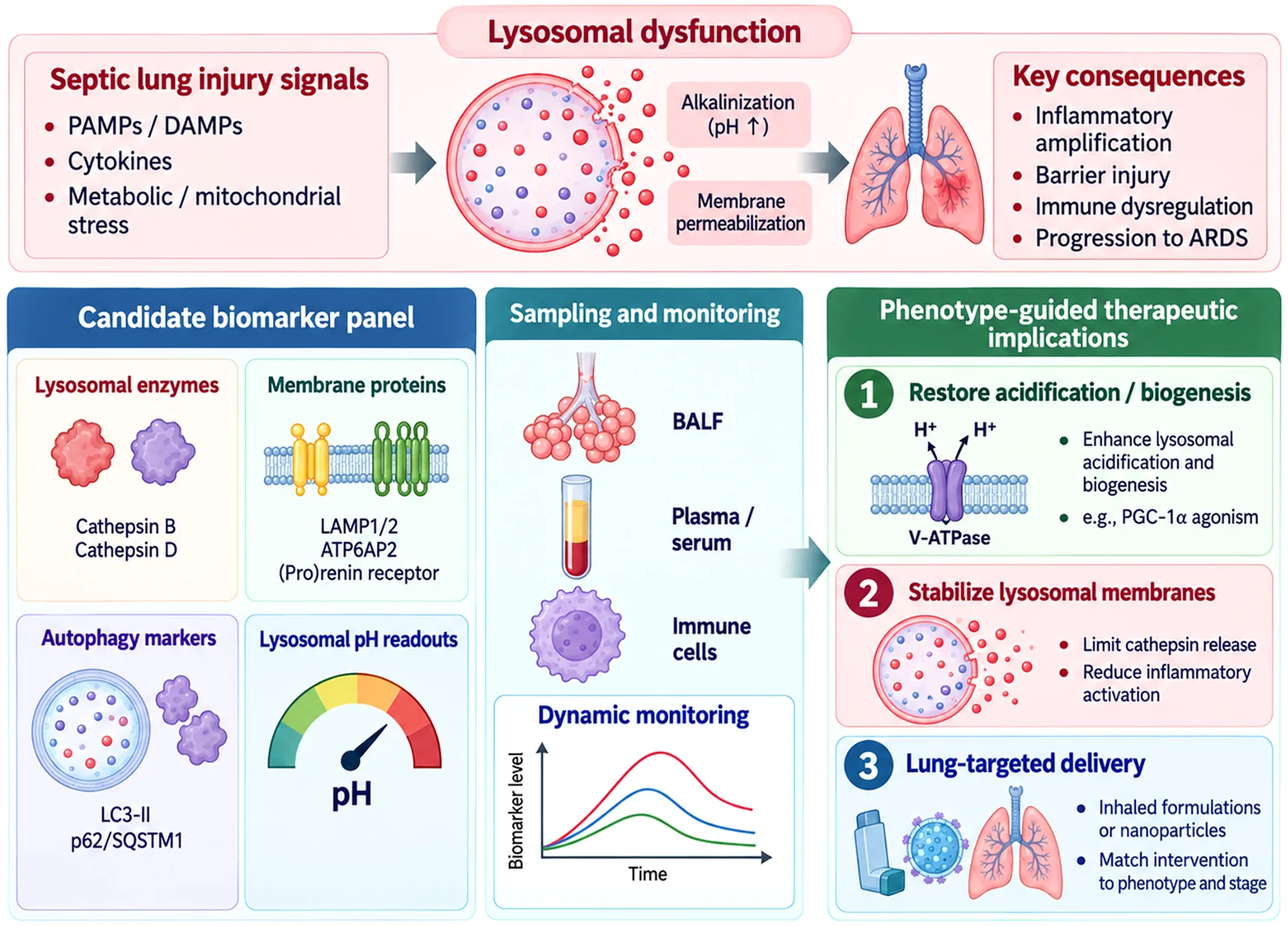
Phenotype-resolved candidate lysosomal biomarker panels in sepsis-associated lung injury. Candidate biomarker strategies should distinguish an acidification axis from a membrane-injury axis. A BALF-focused panel combines calibrated lysosomal pH and intralysosomal cathepsin activity in recovered alveolar macrophages with cytosolic cathepsin redistribution and extracellular cathepsin/LAMP1/2. A peripheral-blood panel combines monocyte lysosomal pH, cathepsin activity/localization, and LC3-II/p62-based flux assessment. These panels are hypothesis-generating and require longitudinal analytical and clinical validation before use for risk stratification or treatment selection.

Accordingly, candidate biomarkers should support stratification only after analytical and prospective clinical validation. The immediate goal is to distinguish reversible acidification failure from membrane-disruptive injury and generalized organelle stress, rather than to define a sepsis-specific lysosomal biomarker from concentration measurements alone.

### 7.2 Therapeutic strategies and clinical implications

A rational therapeutic strategy should be stage- and phenotype-guided rather than aimed at indiscriminate maximal acidification (**Figure 6**). During early or amplification phases, a demonstrable alkalinization-dominant phenotype with impaired phagocyte degradation would justify testing controlled restoration of acidification or lysosomal biogenesis. ZLN005 increased lysosomal acidity and biogenesis in phagocytes and improved bacterial killing and survival in experimental polymicrobial sepsis, but its broad PGC-1α/PI3K/TFEB effects prevent attribution of benefit solely to pH correction (Suzuki et al., 2023). Selective clinically applicable V-ATPase activators are not established.

**Figure 6:**
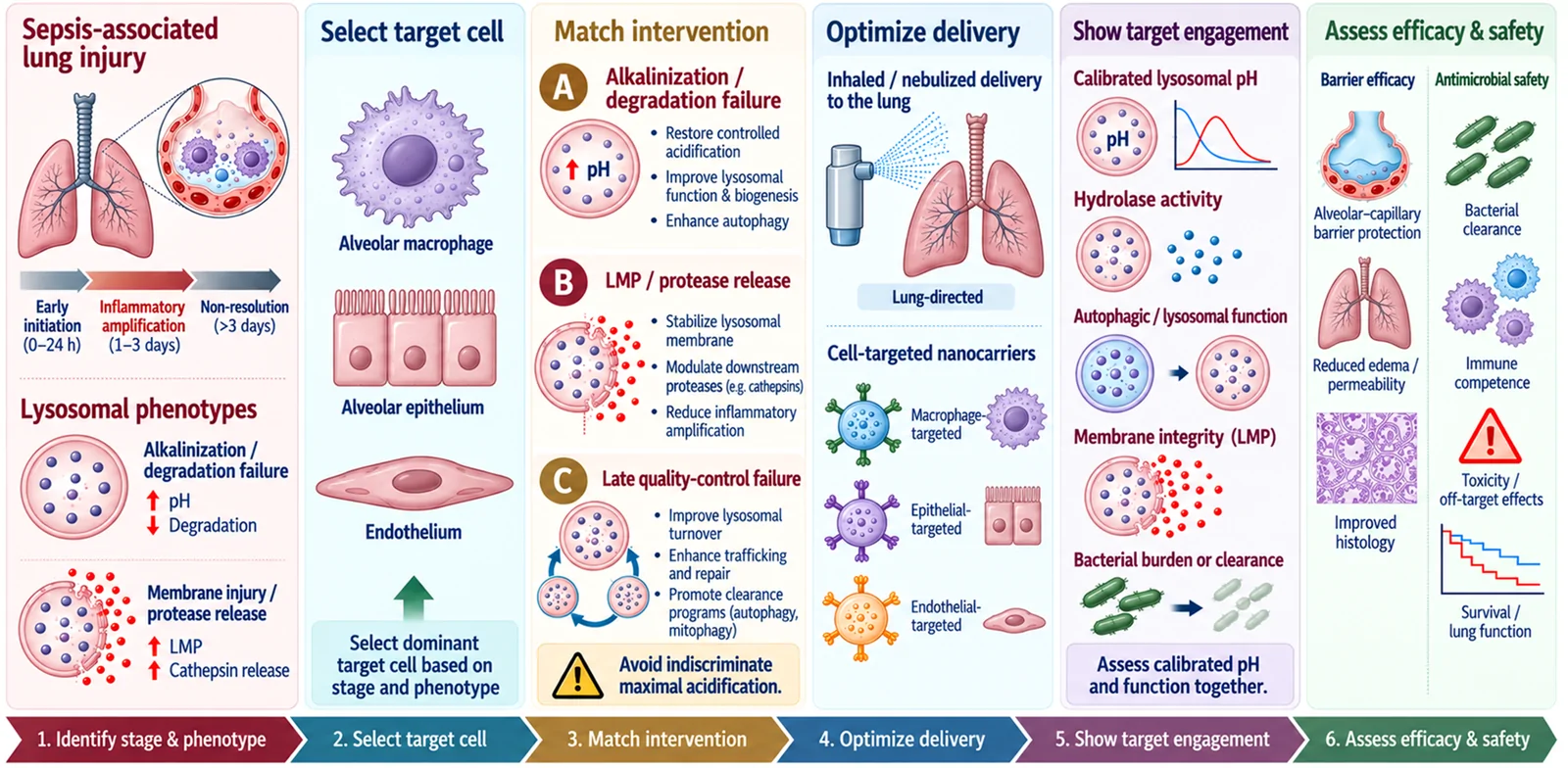
Stage- and phenotype-guided roadmap for lysosome-directed therapy in sepsis-associated lung injury. The proposed roadmap begins with disease-phase and lysosomal-phenotype stratification, followed by selection of the dominant target cell and matching of intervention to the lysosomal phenotype. Within the intervention step, **(A)** alkalinization/degradation failure supports controlled restoration of acidification and lysosomal function, **(B)** lysosomal membrane permeabilization (LMP)/protease release supports membrane stabilization and carefully titrated downstream protease modulation, and **(C)** late quality-control failure supports enhancement of lysosomal turnover, trafficking, and clearance programs. Candidate delivery strategies include inhaled or nebulized lung-directed delivery and cell-targeted nanocarriers. Target engagement should be assessed using calibrated lysosomal pH together with hydrolase activity, autophagic/lysosomal function, membrane integrity, and bacterial burden or clearance. Efficacy and safety evaluation should include alveolar–capillary barrier protection, edema/permeability, histology, bacterial clearance, immune competence, toxicity/off-target effects, survival, and lung function. This is a testable translational roadmap rather than an established clinical treatment algorithm.

A membrane-injury-dominant phenotype, characterized by cathepsin redistribution or release without clear evidence of alkalinization, would instead support testing lysosomal-membrane stabilization or carefully titrated downstream protease modulation. Broad cathepsin inhibition is unlikely to be universally appropriate because lysosomal proteases contribute to microbial killing and tissue remodeling. Treatment selection should therefore be linked to a measurable lysosomal phenotype and disease phase rather than to a single drug class.

Delivery and trial design are equally important. Inhaled formulations, biodegradable nanoparticles, macrophage-targeted carriers, or endothelial-targeted systems may improve local exposure, but edema, heterogeneous ventilation, surfactant changes, inflammatory debris, and phagocyte uptake can alter deposition and retention (Lee et al., 2024; Dhege et al., 2024; Cojocaru et al., 2024). We propose alveolar–capillary barrier injury or permeability as the primary efficacy endpoint in preclinical lung-focused studies, accompanied by bacterial burden/clearance, cell-specific lysosomal pH trajectory, immune competence, toxicity, and survival as mechanistic and safety endpoints. Dose–response and timing studies should define a therapeutic window in which lysosomal function is restored without impairing host defense.

## 8 Discussion

The central conclusion of this Review is that lysosomal pH dysregulation is better supported as a candidate, context-dependent amplifier of infection-associated stress than as a universal initiating lesion. The evidence map makes this boundary explicit: among 21 formally appraised mechanistic axes, 2 (9.5%) are Level I and 3 (14.3%) Level II, whereas 11 (52.4%) are Level III and 5 (23.8%) Level IV (**Table 1**). Thus, the conceptual value of the hub model lies in organizing testable relationships while preserving the distinction between direct septic-lung evidence and mechanistic extrapolation.

### 8.1 Established evidence and causal boundaries

The strongest evidence supports relationships between organelle acidification and phagocyte antimicrobial degradation and between lysosomal/late-endosomal dysfunction, defective autophagic clearance, and experimental septic lung injury (Di et al., 2006; Suzuki et al., 2023; Liu Q. et al., 2021; Jiang et al., 2024; Mo et al., 2018). Evidence is substantially weaker for a single pH defect acting across all pulmonary cell types. ClC-7 regulates luminal chloride and hydrolase function more consistently than bulk acidity, whereas TMEM175 ion selectivity and pulmonary relevance remain contested (Wu et al., 2023; Hu et al., 2022; Riederer et al., 2026). These distinctions define a causal boundary: lysosomal pH dysregulation may amplify injury in selected contexts but should not be presented as a universal upstream driver.

### 8.2 Stage-specific interpretation

The stage-specific model in **Figure 3** provides a temporal scaffold for interpretation. Early septic stress may sensitize lysosomal ion balance and membrane integrity; amplification can involve impaired degradation, membrane permeabilization, inflammasome activation, mitochondrial injury, and pathogen-clearance defects; and persistent dysfunction may contribute to defective immune recovery and tissue repair. These phases are not mutually exclusive, and human studies are needed to determine whether measurable lysosomal phenotypes define clinically useful temporal or biological subgroups.

### 8.3 Hypotheses and open questions

Several mechanisms are retained because they generate specific, testable questions rather than because they are established in septic lung injury. These include TMEM175-dependent ion conductance, proposed IL-6–STAT3–NHE6 regulation, miR-155 effects on lysosomal ion transporters, pH-dependent EV cargo triage, and cell-specific mitochondria–lysosome contact-site regulation. Each requires direct pH manipulation or calibrated measurement in septic lung-relevant cells before it can be incorporated into the causal core of the model. Specifically, it remains unclear whether septic stress alters the abundance, duration, or proton-transfer capacity of mitochondria–lysosome contacts to an extent sufficient to contribute to lysosomal pH dysregulation in lung-resident cells.

### 8.4 Future priorities

Future studies should pair single-cell or spatial identification of lysosomal-stress signatures with calibrated pH measurements, hydrolase activity, membrane-permeabilization assays, and autophagic-flux measurements. Alveolar epithelial–endothelial–macrophage co-culture systems, organoids, precision-cut lung slices, and infection models can help establish cell-specific causality before clinical translation.

Priority questions are which septic signals reproducibly alter lysosomal pH in human lung-relevant cells; whether ClC-7 and TMEM175 act as pH regulators, ion-balance modifiers, or compensatory mechanisms; which biomarker combinations identify reversible dysfunction; and whether phenotype-guided lysosomal interventions improve barrier repair and pathogen clearance without worsening immunosuppression. The therapeutic roadmap in **Figure 6** is intended to make these questions experimentally falsifiable. For mitochondria–lysosome contacts specifically, simultaneous contact-site imaging and calibrated lysosomal pH measurements, combined with perturbation of contact formation and/or mitochondrial electron transport, should test whether contact remodeling causally contributes to pH dysregulation in septic lung-relevant cells.

### 8.5 Limitations of this review

This Review has several limitations. Human studies directly measuring lysosomal pH in sepsis-associated lung injury remain scarce, and experimental studies use heterogeneous lysosomal, endosomal, phagosomal, and lamellar-body readouts that are not directly interchangeable. Several pathways are extrapolated from non-septic or non-pulmonary models. In addition, this is a narrative rather than a systematic review. The original search log was not prospectively preserved, formal risk-of-bias assessment was not performed, and exact PRISMA screening counts cannot be reconstructed reliably; publication and selection bias therefore remain possible. These limitations reinforce the need for calibrated compartment-specific measurements, transparent evidence labeling, and prospective human validation. An additional limitation is that none of the cited studies on sepsis-associated lung injury simultaneously quantified mitochondria–lysosome contacts and lysosomal pH. Accordingly, the relative contributions of V-ATPase/counterion dysfunction and contact-site-mediated proton transfer to lysosomal pH dysregulation remain unresolved in this disease context.

## 9 Conclusion

Lysosomal pH dysregulation is best interpreted as a candidate, context-dependent signal-amplifying hub linking infection-associated inflammatory, metabolic, and mitochondrial stress to selected forms of lung-cell dysfunction. Perturbation of V-ATPase-dependent acidification, ion homeostasis, membrane stability, and autophagy–lysosome flux may impair pathogen clearance and cellular quality control while facilitating cathepsin redistribution, inflammasome activation, regulated cell death, and alveolar–capillary barrier injury.

Clinical translation remains limited by compartment ambiguity, cell- and stage-specific heterogeneity, contested ion-channel mechanisms, and the absence of validated human pH trajectories or bedside assays. The next steps are direct calibrated pH measurements in human lung-relevant cells, causal cell-selective interventions in infection models, longitudinal phenotype-resolved biomarker panels, and therapies designed to restore appropriate lysosomal function without compromising antimicrobial defense. Lysosomal pH homeostasis should therefore be developed as a candidate mechanistic and stratification axis, not as an established precision-medicine target.
