# Supplementary material for: Lysosomal pH dysregulation as a candidate signal-amplifying hub in sepsis-associated lung injury
Source: Front Cell Infect Microbiol. 2026 Oct 20;16:1953667. doi: 10.3389/fcimb.2026.1953667 (PMC13728857; doi:10.3389/fcimb.2026.1953667)
Supplement: Supplementary file 1 [file Table1.docx]

**Supplementary Table S1. Detailed evidence hierarchy linking lysosomal pH dysregulation and related lysosomal processes to sepsis-associated lung injury**

**Evidence-map summary:** 21 mechanistic axes were formally appraised: Level I, 2/21 (9.5%); Level II, 3/21 (14.3%); Level III, 11/21 (52.4%); and Level IV, 5/21 (23.8%). Levels refer to the strength and disease-context specificity of evidence, not to biological importance.

**Compartment/readout note:** “Direct organelle pH measurement” is reported with the relevant compartment (canonical lysosome, endosome, phagosome/phagolysosome, or lysosome-related organelle). Changes in LC3-II, p62/SQSTM1, LysoTracker intensity, cathepsin abundance/redistribution, membrane permeabilization, or hydrolase activity are not treated as stand-alone proof of lysosomal alkalinization.

| **Mechanistic axis** | **Experimental context and principal cell type** | **Primary lysosomal/endolysosomal readout** | **Direct organelle pH measurement (compartment)** | **Causal perturbation** | **Principal finding and relevance to sepsis-associated lung injury** | **Evidence level and principal limitation** | **Reference(s)** |
| --- | --- | --- | --- | --- | --- | --- | --- |
| Circulating mtDNA–STING–TBK1 signaling | Sepsis and circulating-mtDNA mouse models; macrophage-centered innate immune response | Lysosomal acidification, autophagic flux, LC3 and p62-related changes | Yes | STING knockout; STING/TBK1 pathway modulation; exogenous mtDNA | Excessive mtDNA–STING signaling impaired lysosomal acidification and blocked autophagic degradation, thereby promoting persistent inflammatory signaling and lung injury. The study provides the most direct evidence connecting septic signaling, lysosomal pH dysfunction, and acute lung injury. | **Level I. Direct pH-related and causal evidence in sepsis-related lung injury. The precise contribution of individual lung-resident cell populations requires further resolution.** | Liu et al., 2021 (Liu Q. et al., 2021) |
| PGC-1α–TFEB–PI3K/V-ATPase response to ZLN005 | Cecal ligation and puncture polymicrobial sepsis; peritoneal phagocytes; LPS-stimulated THP-1 cells | Lysosomal acidity and mass, TFEB nuclear translocation, DQ-BSA proteolysis, bacterial degradation | Yes | ZLN005 treatment; PGC-1α knockdown; PI3K inhibition with wortmannin | ZLN005 increased lysosomal acidity, lysosomal biogenesis and proteolytic degradation, enhanced bacterial killing, reduced bacterial burden and improved survival. PI3K inhibition prevented ZLN005-induced acidification, supporting a causal PI3K–V-ATPase-associated mechanism. | **Level I. Direct pH measurement and intervention in experimental sepsis, but the study was systemic and phagocyte-focused rather than specific to lung-resident cells or lung injury.** | Suzuki et al., 2023 |
| PICK1-dependent lysosomal competence and autophagic flux | CLP-induced sepsis-associated acute lung injury; wild-type and PICK1-deficient mice | Lysosomal degradative function, LC3-II/LC3-I, p62 and autophagic flux | No | Genetic PICK1 deficiency; pharmacological assessment of autophagy-related signaling | PICK1 deficiency impaired lysosome-dependent autophagic degradation and aggravated pulmonary inflammation, edema and permeability. The results support a causal role for lysosomal impairment in septic lung injury. | **Level II. Direct septic-lung evidence for lysosomal dysfunction, but lysosomal pH was not quantitatively measured; PICK1 may affect several trafficking and signaling processes independently of pH.** | Mo et al., 2018 |
| SIRT1–Rab7-dependent late-endosomal mitophagy | CLP mice and LPS-stimulated pulmonary vascular endothelial cells | Rab7 recruitment, mitophagic flux, p62, damaged mitochondria, mtROS and cytosolic mtDNA | No | SIRT1 inhibition or knockdown; SIRT1 activation or overexpression; Rab7 manipulation; STING and NLRP3 inhibition | SIRT1 promoted Rab7-dependent delivery of damaged mitochondria to degradative compartments. Loss of this pathway caused mtROS and mtDNA accumulation, STING/NLRP3 activation, endothelial inflammation and barrier leakage. | **Level II. Strong causal evidence for late-endosomal/lysosomal trafficking in septic pulmonary endothelium, but the study did not establish lysosomal alkalinization as the initiating defect.** | Jiang et al., 2024 |
| Cathepsin B–inflammasome–pyroptosis signaling | CLP-induced sepsis and lung epithelial cell injury models | Cathepsin B expression or activity; GSDMD cleavage and pyroptosis-associated markers | No | Cathepsin B inhibition with CA-074 | Cathepsin B inhibition reduced epithelial pyroptosis, systemic inflammation and lung injury and improved survival, supporting cathepsin B as a downstream amplifier of septic lung damage. | **Level II. Direct causal evidence in septic lung injury, but increased cathepsin B does not distinguish lysosomal alkalinization, lysosomal membrane permeabilization, increased expression or extracellular release.** | Zhang et al., 2025 (Zhang X. et al., 2025) |
| ATP6V0D2-dependent autophagosome–lysosome fusion | Macrophages challenged with bacteria; ATP6V0D2-deficient experimental models | Autophagosome–lysosome fusion, bacterial degradation and inflammasome activation | No | Genetic ATP6V0D2 deficiency or restoration | ATP6V0D2 facilitated autophagosome–lysosome fusion, restricted inflammasome activation and improved control of bacterial infection. It supports a macrophage-specific role of V-ATPase-associated proteins beyond proton pumping. | **Level III. Strong mechanistic evidence in macrophage infection models, but not direct evidence of altered basal lysosomal pH or sepsis-associated lung injury.** | Xia et al., 2019 |
| CFTR-dependent phagosomal acidification | Macrophage phagocytosis and bacterial-killing models | Phagosomal pH and bactericidal activity | Yes | CFTR inhibition or deficiency | Impaired CFTR function reduced phagosomal acidification and bacterial killing, establishing that organellar acidification is functionally important for macrophage antimicrobial defense. | **Level III. Direct acidification–function relationship in macrophages, but not a sepsis or septic-lung model and not specific to canonical lysosomes.** | Di et al., 2006 |
| V-ATPase-mediated lamellar-body function | Rat alveolar type II epithelial cells | Lamellar-body calcium handling and surfactant secretion | Not directly established for canonical lysosomes | Pharmacological V-ATPase inhibition | V-ATPase activity regulated lamellar-body calcium dynamics and surfactant secretion, demonstrating an acidification-dependent function of lysosome-related organelles in alveolar epithelial cells. | **Level III. Directly relevant to lung epithelium, but lamellar bodies are lysosome-related organelles rather than canonical lysosomes; no septic injury model was used.** | Chintagari et al., 2010 |
| ClC-7/OSTM1-dependent chloride homeostasis | RAW264.7 macrophages and mature phagolysosomes | Luminal chloride, hydrolase activity, cargo degradation and phagosome resolution | Yes | Genetic deletion of Clcn7 | ClC-7 deletion markedly reduced luminal chloride accumulation and hydrolase activity but had negligible effects on phagosomal acidification. The study argues against portraying ClC-7 as a simple permissive counterion pathway required for proton pumping. | **Level III. High-quality direct ion and pH measurements, but not performed in sepsis or lung-resident cells. The findings mainly establish chloride-dependent enzyme regulation rather than direct pH control.** | Wu et al., 2023; mechanistic context (Kasper et al., 2005; Kornak et al., 2001; Jentsch and Pusch, 2018; Accardi, 2021) |
| TMEM175-mediated lysosomal ion conductance | Heterologous cell systems and neurodegeneration-related models | Lysosomal K⁺ and/or H⁺ conductance, luminal pH and proteolytic function | Yes | TMEM175 knockout, variants, agonists or channel recordings | TMEM175 has been implicated in lysosomal membrane potential and pH stability. Some studies support proton-activated H⁺ conductance, whereas others identify predominantly K⁺ conductance or dispute a physiologically relevant proton-selective leak. | **Level III. Direct lysosomal electrophysiology and pH evidence, but the ion-selectivity model remains contested and has not been tested in septic pulmonary cells.** | Lee et al., 2017; Hu et al., 2022; Riederer et al., 2026; Zheng et al., 2022 |
| AKT-dependent V-ATPase assembly and activation | Amino-acid-starved mammalian fibroblasts and engineered HEK293T cells | V-ATPase assembly and activity; lysosomal acidification | Yes | AKT isoform deletion or expression; pathway inhibition | AKT1 and AKT3 promoted starvation-induced V-ATPase assembly and lysosomal acidification, supporting PI3K–AKT as a context-dependent regulator of lysosomal adaptation. | **Level III. Direct mechanistic evidence, but obtained under nutrient starvation rather than sepsis; it does not demonstrate that AKT universally acidifies lysosomes in pulmonary cells.** | Collins et al., 2020; Ratto et al., 2022 |
| TFEB-driven lysosomal biogenesis and V-ATPase regulation | Multiple non-septic mammalian cell and disease models | TFEB localization, CLEAR-network transcription, lysosomal mass and V-ATPase-related function | Variable across studies | TFEB knockout, overexpression or modulation of mTORC1-dependent phosphorylation | TFEB increases lysosomal and autophagic capacity and can regulate V-ATPase-associated programs in selected contexts. It provides a plausible adaptive response to septic lysosomal stress. | **Level III. Robust general lysosomal biology, but individual acidification genes are regulated in a cell- and stimulus-dependent manner; direct septic-lung validation is lacking.** | Settembre et al., 2011; Martina et al., 2012; Roczniak-Ferguson et al., 2012; Wang et al., 2024 (Wang B. et al., 2024) |
| Mitochondria–lysosome contact-mediated proton transfer | HeLa cells and human senescent IMR90 cells; yeast vacuole models; non-septic cellular systems | Mitochondria–lysosome/vacuole contact proximity, proton flux, lysosomal/vacuolar acidification, content degradation and autophagic flux | Yes (canonical lysosome in mammalian cells; vacuole in yeast) | Engineered mitochondria–lysosome/vacuole linkers; mitochondrial electron-transport perturbation; contact-site manipulation | Proton transfer from mitochondria to lysosomes/vacuoles at membrane contacts supported luminal acidification. Enforced organelle contact enhanced or restored acidification and degradative/autophagic function, identifying a contact-dependent mechanism that may complement canonical V-ATPase/counterion control. | Level III. Direct pH and causal contact-site evidence in non-septic models, including human cells, but no sepsis-associated lung or lung-resident-cell model has established that contact remodeling causes lysosomal pH changes. | Tian et al., 2026; Liu Q. et al., 2026 |
| V-ATPase B2-mediated protection of lung epithelial lysosomes | Lung epithelial cells and oxidative/fibrotic lung-injury models | Lysosomal acidity, proteolysis, lysosomal membrane permeabilization and cathepsin D translocation | Yes | V-ATPase B2 overexpression or suppression | Increasing V-ATPase B2 preserved lysosomal acidity and proteolysis, reduced lysosomal membrane permeabilization and cathepsin D redistribution, and attenuated lung injury and fibrosis. | **Level III. Direct lung-cell evidence for pH–membrane-stability coupling, but derived from oxidative and fibrotic injury rather than sepsis.** | Lee et al., 2022 |
| NHE9–Rab7/Rab27 control of degradative versus secretory cargo fate | Non-septic cell models of multivesicular-body trafficking and exosome production | Endosomal pH, Rab7 activity, lysosomal delivery and extracellular-vesicle release | Yes, for endosomal pH | NHE9 overexpression or depletion; Rab7/Rab27 pathway manipulation | NHE9-mediated endosomal alkalinization impaired Rab7-dependent lysosomal delivery and promoted Rab27-dependent exosome release, supporting a pH-sensitive switch between cargo degradation and extracellular export. | **Level III. Direct endosomal-pH and trafficking evidence, but not a septic or lung-cell model; endosomal alkalinization should not be equated automatically with lysosomal alkalinization.** | Duhaini et al., 2025 |
| Lysosomal impairment–induced mitochondrial cargo release in EVs | Non-septic cellular models with pharmacological or trafficking-associated lysosomal impairment | EV number and size; ubiquitinated proteins, mtDNA and other mitochondrial cargo within EVs | Not necessarily quantified as pH | Pharmacological inhibition of lysosomal acidification; disruption of Rab7-dependent trafficking | When lysosomal degradation was impaired, cells increased the extracellular release of vesicles containing mitochondrial and ubiquitinated cargo, supporting competition between lysosomal clearance and extracellular disposal. | **Level III. Strong cargo-fate evidence, but the models were not septic lung cells and active EV secretion must be distinguished from passive release caused by cell injury.** | Liang et al., 2023 |
| Alveolar epithelial cell-to-macrophage EV signaling | Sepsis-induced acute lung injury models; alveolar epithelial cells and alveolar macrophages | EV abundance and cargo, including miR-92a-3p, tenascin-C and lncRNA Rmrp | No | EV inhibition or transfer; cargo-specific gain- and loss-of-function approaches | Epithelial EV cargo promoted macrophage PI3K–AKT–NF-κB activation or pyroptosis during inflammatory phases, whereas lncRNA Rmrp promoted macrophage metabolic suppression and immune tolerance during later immunosuppression. | **Level IV. The intercellular communication pathways are supported in septic lung injury, but no study directly demonstrated that lysosomal pH altered EV biogenesis, cargo selection or recipient-cell responses.** | Liu et al., 2021 (Liu F. et al., 2021); Gong et al., 2024; Liu et al., 2026 |
| Macrophage-to-epithelial or endothelial inflammatory communication | Sepsis-induced lung injury; macrophages, alveolar epithelial cells and pulmonary vascular endothelial cells | EV cargo or pyroptosis-associated mediators, including CD13, GBP2 and ZBP1-related signals | No | EV-transfer experiments; cargo knockdown or inhibition; cell-death pathway manipulation | Macrophage-derived CD13 promoted epithelial necroptosis, macrophage EV-associated GBP2 promoted endothelial ferroptosis, and ZBP1-associated macrophage pyroptosis impaired epithelial mitochondrial and barrier function. | **Level IV. Direct communication and injury mechanisms are established, but the proposed upstream role of macrophage lysosomal alkalinization in cargo export remains untested.** | Gong et al., 2022; Zhou et al., 2026; Li et al., 2025 (Li Z. et al., 2025) |
| Endothelial cell-to-myeloid-cell EV signaling | Experimental sepsis-related lung injury; endothelial cells, monocytes and neutrophils | Endothelial EV release and surface or intravesicular cargo, including VCAM1 and KPNB1 | No | EV depletion or transfer; receptor- or cargo-specific inhibition | Endothelial EVs reprogrammed monocytes through VCAM1–ITGA4 signaling and promoted aberrant neutrophil trafficking through KPNB1-associated mechanisms. | **Level IV. Direct septic communication evidence, but endothelial lysosomal pH, EV cargo triage and lysosomal degradation were not measured.** | Wang et al., 2024 (Wang L. et al., 2024); Zi et al., 2024 |
| Neutrophil-derived NET and DLL4 signaling to pulmonary endothelium | Sepsis-associated lung injury; neutrophils and pulmonary endothelial cells | NET-derived DNA and proteins; endothelial STING, junctional proteins and PANoptosis pathways | No | NET degradation or inhibition; STING or pathway inhibition; DLL4–Notch1 manipulation | NETs promoted STING-dependent immunothrombosis and endothelial junction loss, whereas DLL4-positive neutrophils induced Notch1/ZBP1-associated endothelial PANoptosis. | **Level IV. Strong evidence for neutrophil–endothelial communication, but lysosomal pH was neither measured nor shown to initiate NET generation or endothelial injury.** | Zhu et al., 2023; Hong et al., 2026; Jin et al., 2025 |
| NLRC4-associated macrophage–fibroblast inflammatory network | Experimental acute respiratory failure and lung injury; pulmonary fibroblasts and macrophages | NLRC4 inflammasome activation, caspase-1, GSDMD and pyroptosis | No | Genetic or pharmacological disruption of NLRC4-associated pyroptosis | Pyroptosis in both fibroblasts and macrophages amplified inflammation, barrier failure and respiratory dysfunction, demonstrating that fibroblasts participate actively in acute inflammatory communication. | **Level IV. Direct multicellular inflammatory evidence, but no causal connection to lysosomal pH or lysosomal membrane injury was tested.** | Zhang et al., 2025 (Zhang Y. et al., 2025) |

**Evidence-level definitions.** Level I: direct organelle acidity/pH-related measurement together with causal intervention in sepsis or sepsis-associated lung injury. Level II: causal lysosomal, late-endosomal, or autophagy–lysosome evidence in septic lung injury without direct pH measurement. Level III: direct mechanistic evidence for pH, ion conductance, degradation, cargo trafficking, or organelle-contact-mediated proton transfer in non-septic models. Level IV: an intercellular communication pathway is established in septic lung injury, but dependence on lysosomal/endolysosomal pH remains hypothetical.

**Supplementary Table S2. Intercellular communication pathways and candidate lysosomal/endolysosomal pH-dependent checkpoints in the septic lung**

**Interpretive note:** The communication axes summarized below are supported in experimental sepsis or sepsis-associated lung injury, but the proposed pH-dependent checkpoints are hypothesis-generating (Level IV) unless explicitly stated otherwise. None of the cited communication studies directly established that changing lysosomal/endolysosomal pH alters extracellular-vesicle abundance or cargo, recipient-cell cargo processing, NET persistence, or the strength of the indicated communication pathway. Active vesicle secretion should also be distinguished from passive cargo release caused by cell lysis.

| **Communication axis** | **Extracellular signal or cargo** | **Recipient-cell signaling and response** | **Consequence in the septic lung** | **Likely disease phase** | **Candidate lysosomal/endolysosomal checkpoint** | **Evidence status and major limitation** | **Reference(s)** |
| --- | --- | --- | --- | --- | --- | --- | --- |
| Alveolar epithelial cell → alveolar macrophage | Epithelial cell-derived exosomal miR-92a-3p | miR-92a-3p suppresses PTEN and enhances PI3K–AKT–NF-κB signaling in alveolar macrophages, promoting macrophage activation and inflammatory mediator production. | Amplification of alveolar inflammation and lung injury | Early hyperinflammatory and amplification phases | In sender cells, impaired endolysosomal acidification may reduce degradation of miRNA-containing MVB cargo and favor Rab27-dependent EV release. In recipient macrophages, endolysosomal pH may influence EV processing, intracellular cargo release and the duration of inflammatory signaling. | **The epithelial cell–macrophage EV pathway is supported in sepsis-induced acute lung injury, but lysosomal pH, EV cargo degradation and pH-dependent cargo release were not measured. The proposed pH checkpoint is inferred from non-septic cargo-trafficking studies (Rizzollo and Agostinis, 2025; Duhaini et al., 2025).** | Liu F. et al., 2021; mechanistic context (Duhaini et al., 2025; Liang et al., 2023) |
| Stressed alveolar epithelial cell → alveolar macrophage | Tenascin-C-enriched exosomes | Exosomal tenascin-C engages macrophage TLR4, increases mitochondrial ROS and promotes macrophage inflammasome activation and pyroptosis. | Exaggerated macrophage pyroptosis and aberrant inflammatory amplification | Early hyperinflammatory and amplification phases | Reduced lysosomal acidification in epithelial cells may shift tenascin-C-containing MVBs from lysosomal degradation toward extracellular release. In macrophages, impaired lysosomal and mitophagic quality control may increase mtROS accumulation and lower the threshold for pyroptosis. | **Direct evidence supports tenascin-C-mediated epithelial–macrophage communication, but no study has shown that lysosomal alkalinization controls exosomal tenascin-C loading, secretion or macrophage uptake.** | Gong et al., 2024; mechanistic context (Duhaini et al., 2025; Liang et al., 2023; Lee et al., 2022) |
| Alveolar macrophage → lung epithelial cell | Macrophage-derived exosomal aminopeptidase N/CD13 | Exosomal CD13 activates epithelial TLR4, inducing ROS production, mitochondrial dysfunction, NF-κB activation and RIPK1–RIPK3-dependent necroptosis. | Epithelial cell loss, impaired alveolar integrity and increased lung injury | Inflammatory amplification phase | Macrophage lysosomal alkalinization could impair degradation of inflammatory membrane proteins and favor their incorporation into EVs. In epithelial recipient cells, defective lysosomal acidification and mitophagy may impair removal of damaged mitochondria and increase susceptibility to necroptosis. | **The CD13–TLR4–necroptosis pathway is causally supported, but lysosomal pH was not measured in either sender or recipient cells. It remains unknown whether pH restoration changes EV-associated CD13 abundance.** | Gong et al., 2022; mechanistic context (Duhaini et al., 2025; Liang et al., 2023; Lee et al., 2022) |
| Pyroptosis-associated alveolar macrophage → alveolar type II epithelial cell | ZBP1-dependent macrophage pyroptosis-associated inflammatory mediators and DAMPs; the decisive extracellular mediator has not been fully defined | ZBP1 activation promotes inflammasome assembly and macrophage pyroptosis. Released inflammatory signals impair mitochondrial function and barrier-related activity in alveolar type II epithelial cells. | Mitochondrial dysfunction, epithelial injury and alveolar barrier failure | Hyperinflammatory and amplification phases | Macrophage lysosomal dysfunction may promote accumulation of damaged mitochondria, mtROS and inflammasome-activating signals, thereby facilitating pyroptosis and DAMP release. Recipient-cell lysosomal and mitophagic capacity may determine epithelial sensitivity to transferred inflammatory stress. | **Single-cell and functional evidence supports a pathogenic ZBP1-associated macrophage subset. However, this is not a defined EV pathway, the dominant extracellular mediator remains incompletely resolved, and lysosomal pH dependence has not been tested.** | Zhou et al., 2026 |
| Alveolar type II epithelial cell → alveolar macrophage | Exosomal lncRNA Rmrp | Rmrp suppresses macrophage glycolysis and promotes an immune-tolerant or immunosuppressive macrophage phenotype. | Reduced macrophage antimicrobial and inflammatory competence, potentially contributing to secondary immunosuppression | Late or immunosuppressive phase | Stage-dependent changes in epithelial endolysosomal pH may affect lncRNA degradation, MVB trafficking and exosomal loading. In recipient macrophages, lysosomal pH may interact with metabolic reprogramming, autophagic flux and pathogen-processing capacity. | **The Rmrp-mediated immunosuppressive pathway is supported in sepsis models, but no direct evidence links lysosomal pH to Rmrp loading, exosome secretion or macrophage metabolic reprogramming.** | (Liu et al., 2026), mechanistic context (Duhaini et al., 2025; Liang et al., 2023) |
| Macrophage → pulmonary vascular endothelial cell | Macrophage-derived EV-associated GBP2 | EV-delivered GBP2 interacts with endothelial OTUD5, promotes GPX4 ubiquitination and depletion, and induces endothelial ferroptosis. | Pulmonary endothelial death, increased vascular permeability and alveolar–capillary barrier disruption | Inflammatory amplification phase | In macrophages, defective acidification may divert GBP2-containing cargo away from lysosomal degradation and toward EV export. In endothelial cells, impaired lysosomal and mitophagic quality control may increase mtROS, labile iron and lipid peroxidation, thereby lowering the ferroptosis threshold. | **The EV-GBP2–OTUD5–GPX4 pathway is directly supported in sepsis-induced acute lung injury. However, the proposed sequence from macrophage lysosomal alkalinization to EV-associated GBP2 release has not been demonstrated.** | Li Z. et al., 2025; mechanistic context (Jiang et al., 2024; Duhaini et al., 2025; Liang et al., 2023) |
| Pulmonary endothelial cell → circulating monocyte | Endothelial cell-derived EVs bearing surface VCAM1 | EV-associated VCAM1 binds monocyte ITGA4, activates NF-κB signaling and reprograms monocyte differentiation and inflammatory activity. | Recruitment and reprogramming of inflammatory monocytes, promoting sustained pulmonary inflammation | Early inflammatory and amplification phases | Endolysosomal trafficking and pH in endothelial cells may regulate MVB fate, EV abundance and the sorting or surface retention of VCAM1. Recipient-monocyte lysosomal competence may affect EV processing and the duration of NF-κB activation. | **Direct evidence supports VCAM1-dependent endothelial EV–monocyte communication. However, endothelial or monocyte lysosomal pH was not measured, and a pH-dependent effect on VCAM1 sorting remains hypothetical.** | Wang L. et al., 2024; mechanistic context (Duhaini et al., 2025; Liang et al., 2023) |
| Pulmonary endothelial cell → neutrophil | KPNB1-enriched endothelial cell-derived EVs | Endothelial EV cargo alters neutrophil trafficking and promotes reverse transendothelial migration. | Aberrant neutrophil redistribution and subsequent remote lung or organ injury | Amplification and systemic propagation phases | Altered endothelial endolysosomal pH may change KPNB1 cargo sorting and EV secretion. In neutrophils, endolysosomal processing may influence EV-cargo release and migratory reprogramming. | **The endothelial EV–neutrophil trafficking pathway is supported experimentally, but neither lysosomal pH nor pH-dependent KPNB1 sorting was assessed. The mechanism linking EV uptake to neutrophil reverse migration requires further definition.** | Zi et al., 2024; mechanistic context (Duhaini et al., 2025; Liang et al., 2023) |
| Neutrophil → pulmonary endothelial cell | NET-derived extracellular DNA, histones, myeloperoxidase and neutrophil elastase | NET-derived DNA activates endothelial STING signaling and promotes tissue-factor expression and immunothrombosis. | Pulmonary microvascular thrombosis, endothelial activation and impaired perfusion | Hyperinflammatory and immunothrombotic phases | Recipient endothelial lysosomal dysfunction may impair nucleic-acid clearance and prolong STING activation. Macrophage phagolysosomal alkalinization may delay NET and apoptotic-neutrophil clearance, extending extracellular DAMP exposure. | **The NET–STING–immunothrombosis pathway is supported in sepsis-associated lung injury. However, lysosomal alkalinization has not been shown to initiate NET formation or STING activation in this setting. The most plausible pH-dependent role is defective signal clearance rather than signal generation.** | (Zhu et al., 2023), mechanistic context (Liu Q. et al., 2021; Weiss and Schaible, 2015; Watts, 2022; Nguyen and Yates, 2021) |
| Neutrophil → pulmonary endothelial cell | NET-associated DNA, histones and proteases | NETs activate endothelial Wnt7a–β-catenin–HDAC5 signaling and reduce claudin-5, ZO-1 and occludin expression. | Pulmonary endothelial tight-junction disruption and microvascular leakage | Inflammatory amplification phase | Impaired macrophage lysosomal acidification may delay NET clearance. In endothelial cells, defective lysosomal degradation and mitochondrial quality control may amplify oxidative stress and interfere with junctional-protein turnover and repair. | **Direct evidence supports NET-induced tight-junction injury, but the involvement of lysosomal pH is indirect. It remains unknown whether restoring macrophage or endothelial lysosomal acidity accelerates NET clearance or preserves junctional proteins.** | Hong et al., 2026 |
| eCIRP-induced DLL4-positive neutrophil → pulmonary vascular endothelial cell | Membrane-associated DLL4 engaging endothelial Notch1 | DLL4–Notch1 signaling activates a ZBP1-associated PANoptotic program in endothelial cells. | Endothelial PANoptosis, barrier failure and aggravated septic lung injury | Hyperinflammatory and amplification phases | Lysosomal and mitophagic dysfunction in endothelial cells may increase mitochondrial stress and lower the threshold for integrated inflammatory cell death. Endolysosomal trafficking could also affect Notch receptor processing, but this has not been tested in septic endothelium. | **The DLL4–Notch1–ZBP1/PANoptosis pathway is causally supported. There is no direct evidence that lysosomal pH controls DLL4 expression, Notch1 activation or endothelial PANoptosis in this model.** | Jin et al., 2025 |
| Pulmonary fibroblast–macrophage inflammatory coupling | Pyroptosis-associated IL-1-family cytokines, DAMPs and matrix-derived inflammatory fragments; a single directional ligand–receptor pair has not been defined | NAIP–NLRC4 inflammasome activation induces caspase-1- and GSDMD-dependent pyroptosis in both pulmonary fibroblasts and macrophages. Pyroptotic products may reciprocally amplify inflammation in neighboring cells. | Acute inflammation, respiratory dysfunction and barrier injury, with possible consequences for subsequent matrix remodeling | Acute amplification and potentially non-resolving phases | Lysosomal alkalinization may impair intracellular degradation and extracellular clearance of DAMPs and matrix fragments, prolonging fibroblast–macrophage inflammatory coupling. Lysosomal membrane permeabilization may also facilitate inflammatory cell death, but this has not been established for NLRC4 activation. | **The study demonstrates multicellular participation of fibroblasts and macrophages rather than a fully resolved directional communication pathway. Lysosomal pH was not measured and should not be presented as an initiator of NLRC4 activation.** | Zhang Y. et al., 2025 |
